# Filtration evaluation and clinical use of expired elastomeric P-100 filter cartridges during the COVID-19 pandemic

**DOI:** 10.1017/ice.2020.257

**Published:** 2020-05-27

**Authors:** Harsh H. Patolia, Jin Pan, Charbel Harb, Linsey C. Marr, Anthony W. Baffoe-Bonnie

**Affiliations:** 1 Virginia Tech Carilion School of Medicine, Roanoke, Virginia; 2 Civil and Environmental Engineering, Virginia Tech, Blacksburg, Virginia; 3 Carilion Clinic Roanoke Memorial Hospital, Roanoke, Virginia

*To the Editor—*The limited supply of more conventional disposable personal protective equipment (PPE), namely single-use N95 filtering facepiece respirators (FFRs), among hospital systems in the United States during the COVID-19 pandemic has been alleviated with the adoption of extended use and reuse policies by the Centers for Disease Control and Prevention (CDC).^1^ These measures, along with a variety of implemented decontamination methodologies (eg, ultraviolet germicidal irradiation, vaporized hydrogen peroxide, etc), have prolonged PPE supplies during pressing times. Another strategy adopted by the CDC and health systems to protect healthcare providers caring for COVID-19 patients and patients under investigation in limited resource settings includes the use of elastomeric FFRs with reusable cartridges. Although elastomeric respirators have not been approved by the Food and Drug Administration for fluid resistance, they have been endorsed by the CDC as reasonable alternatives for N95 FFRs during the COVID-19 pandemic due to their filtration approval by the National Institutes for Occupational and Safety Health (NIOSH).^2^ Subsequently, elastomeric respirators have formed a major arm of the COVID-19 pandemic response strategy in many hospital systems.

Elastomeric FFRs contain plastic components that can be easily decontaminated for repeated use. Those elastomeric respirators containing a full facepiece can afford greater protection to wearers by diminishing face seal leakage and provide eye protection. Although various types of elastomeric FFRs exist, their filtration mechanism is universally housed in exchangeable cartridge filters. The replacement of these filters is determined by their respective expiration dates indicated by the manufacturers as well as the presence of visible soiling, contamination, and/or damage. The strategic national stockpile was not significantly restocked after it had been mobilized for the 2009 influenza pandemic.^3^ Thus, cartridges for elastomeric respirators in the strategic national stockpile may have surpassed their expiration dates, which voids NIOSH approval regarding filtration efficiency.

At our institution, we hoped to distribute the P100 elastomeric respirators to providers caring for COVID-19 patients and patients under investigation that we received from the strategic national stockpile. P100 respirators are designed to filter out at least 99.97% of airborne particles. Although data regarding the performance of P100 respirators for viral aerosols are scarce, clear evidence has demonstrated their filtration superiority compared to N95 respirators.^4^ During our initial distribution of P100 elastomeric respirators, we observed that our supply of filter cartridges from the strategic national stockpile had surpassed their 5-year shelf life; they had expired in 2014. They were well kept in unopened packages. Based on collected feedback from frontline providers, many expressed concern and alarm regarding the effectiveness of these devices and their filtering efficiency. In an effort to rapidly verify the filtering efficiency of our elastomeric respirator supply and to ensure the safety of our provider staff, we evaluated a sample of our P100 filter cartridges using quantitative methods.

**Fig. 1.**
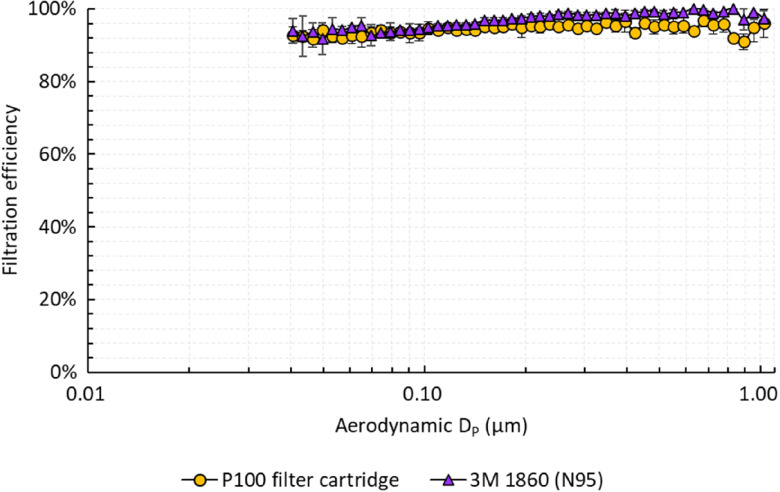
Filtration efficiency of the P100 filter cartridge and 3M 1860 (N95) for salt (NaCl) particles 0.04–1.0 μm in aerodynamic diameter. Error bars represent standard deviations across 2 cartridges, each tested twice.

Filtration efficiency was evaluated by challenging the cartridges with aerosols generated from a 2% sodium chloride solution using a Collison 3-jet nebulizer (BGI MRE-3) in a 280-L polyethylene chamber (Sigma AtmosBag). The concentration and size distribution of the aerosols were measured using a scanning mobility particle sizer (TSI SMPS 3936). For testing, the cartridge was sealed inside a cardboard box, and the cartridge and box were further covered with electrical tape, except for the opening of the cartridge, to prevent leakage through the box itself. A vacuum line was connected to the box, and the challenge aerosol was pulled through the cartridge at a flow rate of 15 L per minute. Figure 1 shows the filtration efficiency of the cartridge compared to that of an N95 that was measured using the same experimental setup. The cartridge removed at least 90% of aerosols over the size range of 0.04–1 µm, including the most penetrating size of 0.1–0.3 µm where it demonstrated an efficiency comparable to the N95 respirator.

Our studies demonstrated that the filtration efficiency of P100 filter cartridges past their 5-year expiration date was not significantly different from that of an N95 respirator (3M 1860) (*P* > .05), although it was <99.97%. These studies are preliminary, but they suggest the possibility of safely implementing expired filter cartridges in elastomeric respirators during limited resource scenarios and patient surges, particularly during the significant logistical challenge presented by the COVID-19 pandemic. With considerable disruptions in global supply chains for N95 respirators, hospital systems may consider adopting guidelines that conditionally endorse the use of expired filter cartridges in elastomeric FFRs.
